# Book Review

**Published:** 1908-06

**Authors:** 


**Acute Pneumonia: its signs, symptoms, and treatment.** By SEYMOUR TAYLOR, M.D. Pp. 64. London: Henry J. Glaisher. 1907.—These post-graduate lectures give the latest ideas on the subject, and are grounded on the view that pneumonia is an infectious disease which calls for fresh air and cleanliness for the benefit of the patient, and for all the sanitary precautions on the part of the nurse, which are taken in the case of a "spreading" disease such as erysipelas, such precautions, in short, as may be termed "medical Listerism."

**Consumption: treatment at home and rules for living.** By H. WARREN CROWE, M.D. Second Edition. Pp. 36. Bristol: John Wright & Co. 1907.—This little book is intended for those consumptives who cannot go to a sanatorium, or who cannot complete their cure there; it gives the ideal treatment, whether in or out of a sanatorium. Experience has proved that the longer the treatment is carried out the more certain and lasting will be the cure; the instructions contained in the book will enable any patient to carry on his cure indefinitely. It should be remembered that at first rest is the cure in combination with fresh air and good food. Exercise becomes needful for the purpose of regaining strength after the cure. Few patients can or will carry out the programme at its best, excepting under the discipline of sanatorium life, but the second best is sometimes the only thing possible, and determination will do a great deal when well directed by a reliable guide such as this.

**Minor Medicine.** By WALTER ESSEX WYNTER, M.D. Pp. x., 275. London: Sidney Appleton. 1907.—This treatise on the nature and treatment of common ailments is designed for the student in order to compensate for the undoubted facts that trifling disorders do not present themselves at a hospital, or are intercepted in the casualty department to spare the time and energy of the visiting staff; hence the present-day student has little or no opportunity of familiarising himself with those slighter maladies, which are likely to be among the first encountered when he commences practice. The seventeen pages of prescriptions at the end of the volume are of especial use in consideration of the ignorance of the student, as well as of many practitioners, in the almost forgotten art of writing a prescription without the aid of the proprietary pharmacist. They are for the most part well tried formulæ, endorsed by the authority of some distinguished specialist.

**The Treatment of Disease in Children.** By G. A. SUTHERLAND, M.D. Pp. viii., 311. London: Hodder & Stroughton. 1907. Dr. Sutherland has written a book which deals as exclusively with treatment as is possible. The other virtues of his work are that it embodies his own experience and ideas without too much reference to those of others; and, further, that his ideas are full of common sense. There are many valuable paragraphs on the hygienic and dietetic treatment of disease in children, and the author makes it clear that he is no blind believer in drugs. Indeed, he appears occasionally to overstep the limit, and to condemn therapeutic methods whose value is beyond doubt. His dogmatism also leads him into statements which are scarcely tenable; for example, he says the colic of early infancy never afflicts properly-fed babies. On page 207 an "incompatible" prescription appears; calcium chloride with magnesium sulphate gives a calcium sulphate precipitate. The book is of undoubted value, in that it is a statement of experience by a physician who has seen a great deal of the diseases of children; but it needs to be read with discrimination on account of the faults pointed out in this review.

**Manual of Practical Anatomy.** By D. J. Cunningham, M.D. Fourth Edition. Two Vols. Edinburgh: Young J. Pentland. 1907.—It is always gratifying to be able to speak in unqualified praise about any subject for review. And we can do this without any hesitation in the present case. For lucidity of description and clearness of illustration it would be difficult to improve upon this little two-volume manual. But since it is only a short time since the appearance of the third edition, and as the present one is essentially the same, it is unnecessary to describe it at any great length. The chief changes and additions relate to the sections on the thorax and abdomen. Some new and most artistic illustrations have been added of the lymphatics of the breast, axilla and Scarpa's triangle, the thorax as seen from each side after the removal of the outer chest wall and the corresponding lung, the heart of a calf showing the moderator band, and of the external aspect of the eye. The division of the stomach into two chambers, with the anatomical distinction of the pyloric vestibule is described and figured. The pelvic fascia has a decidedly improved and simplified description. The account of the female perineum and pelvis still remains much inferior to that of the male, which, considering its greater practical importance, is a matter for regret, especially as this is a fault common to all text-books of anatomy. It is remarkable that a place should still be found for Luschka's picture of the diaphragm, representing it as having ten slips of costal origin on one side. On the whole, the book is perhaps the most lucid account of the essentials of anatomy ever published.

**Surgical Applied Anatomy.** By Sir Fredk. Treves, Bart. Fifth Edition. Edited by Arthur Keith, M.D. Pp. xii., 640. London: Cassell & Company Ltd. 1907.—This most excellent manual has passed through five editions during the last quarter of a century. The fifth edition maintains most of the admirable characteristics of the earlier ones, and sustains its reputation by careful revision and the introduction of much up-to-date material. It is a storehouse of practical information, and is almost a necessity to those revising their studies for the higher examinations. In our opinion it is the best book of its kind, and we would be glad to see the inclusion in the next edition of an adequate description of the surgical anatomy of the uterus and broad ligaments, and illustrations of the lymphatics of the breast and stomach, in accordance with their surgical importance.

**The Pocket Anatomy.** By C. H. Fagge. London: Baillière, Tindall & Cox. 1908.—Our old friend, which we all have secretly trusted and publicly abused, *The Pocket Gray,* now comes to us with a new and more sensible name. Its contents have not much altered, but all the alterations are improvements. The actions of muscles are now inserted in brackets after each one has been described, several of the arteries and nerves are described in a more tabular form. The lower part of the bowel is divided, and named as in all modern books the iliac colon, pelvic colon and rectum, the term sigmoid flexure being dropped. It has always struck us as very curious that in a cram book of this sort the one section of anatomy to be omitted is that on the bones, whilst those on the brain, eye and ear are inserted. The fact that 30,000 have already been issued proves the value which it has proved to many readers.

**Hygiene of the Lung in Health and Disease.** By Prof. Dr. Leopold von Schrötter. Translated by H. W. Armit. Pp. xv., 135. London: Rebman Limited. 1907.—This book was written by the eminent Viennese professor for the purpose of instructing the lay person on the functions of the respiratory organs, and on the importance which a free exercise of these functions possesses. The instruction of the laity will effect hygienic improvements far better than the introduction of new laws and punishments. The knowledge herein contained is calculated to enable the reader to become a useful member of the hygienic world, and to join effectually in the active crusade against that disease which has worked such ravage among men—tuberculosis. This English translation should greatly extend the usefulness of the book, and a wide circulation of it among Englishmen will do much to increase the popularity and utility of our sanatoria for consumptives.

**Tropical Diseases.** By Sir Patrick Manson, K.C.M.G., M.D., LL.D. Pp. xx., 876. London: Cassell & Company Limited. 1907.—To the general practitioner the territory of tropical diseases is a "blue" chamber which from lack of genuine curiosity he rarely enters. There is, however, but little doubt that the study of tropical ailments must in the future claim some of the time of the ever over-burdened medical student. The present advances against the ravages of disease in Africa, South

America and India are all preparing the way for the healthy life of the white man in those countries. By that time this fourth edition of some 900 pages will probably have expanded to a ten-volume system, or a tropical encyclopædia medica. Meanwhile, it behoves teachers of medicine to keep abreast with the demands of the time, and to make arrangements whereby students and practitioners may see imported cases of tropical diseases. A critical review of such a standard book as this is unnecessary. It only remains to say that it has been brought thoroughly up to date, and constitutes an admirable addition to English medical literature.

**The Proceedings of the Royal Society of Medicine.** Vol. I. Nos. I. and II. November and December, 1907. London: Longmans, Green & Co.—The amalgamation of the fifteen medical societies of London, and thereby the formation of the one Royal Society of Medicine with thirteen special sections has necessarily led to a simplification of the reports hitherto published by the fifteen separate societies. An editorial committee has been appointed, and each section has its own editorial representative on that committee. The proceedings will be published monthly, and will contain the papers read at each of the sections during the previous month. The matter is being so arranged that each section can, if desired, have its own portions detached and bound separately at the end of the year. The first two numbers are before us: they are well and carefully printed in excellent, readable type, and they contain several excellent full-page engravings. They are edited by Dr. John Nachbar, M.A., under the direction of the editorial committee. We cannot do otherwise than offer our congratulations to the committee on the form in which the work of the Royal Society of Medicine is being recorded.

**The Romance of Medicine.** By RONALD CAMPBELL MACFIE, M.B. Pp, viii., 312. London: Cassell & Co. Ltd. 1907.—The first two chapters, on the beginnings of medicine, describe a thrice-told story, but go a little further back than the Greeks and Romans, and give a brief account of certain medical papyri discovered in Egypt, and dating back to about 1600 B.C. The times of Hippocrates are well described, and afterwards come Aristotle and Galen, who in Rome did the work which has made him famous to all time. After a rapid survey of the Middle Ages, the renaissance of Greek learning in Europe, the revival of anatomy and the work of Harvey, we are told that "man is not only a menagerie, he is also an aquarium. His ancestor was probably a cell that floated in the circumpolar seas, and some of his cells still prefer a marine and briny habitat." This brings us on to the work of Virchow and of Metchnikoff, who is described as the greatest of living scientists. The cell and the microbe are evolutions of quite modern times. The story is quite an enchanting one, and will well repay something more than a cursory review.

